# Influence of Ivabradine on the Anticonvulsant Action of Four Classical Antiepileptic Drugs Against Maximal Electroshock-Induced Seizures in Mice

**DOI:** 10.1007/s11064-016-2136-1

**Published:** 2017-01-12

**Authors:** Katarzyna M. Sawicka, Agnieszka Wawryniuk, Agnieszka Zwolak, Jadwiga Daniluk, Monika Szpringer, Magdalena Florek-Luszczki, Bartlomiej Drop, Dorota Zolkowska, Jarogniew J. Luszczki

**Affiliations:** 1 Medical University in Lublin, Lublin, Poland; 2 The Jan Kochanowski University in Kielce, Kielce, Poland; 3 Institute of Rural Health, Lublin, Poland; 4 School of Medicine, University of California–Davis, Sacramento, California USA

**Keywords:** Antiepileptic drugs, Ivabradine, Maximal electroshock-induced seizures, Pharmacokinetic/pharmacodynamic interaction

## Abstract

Although the role of hyperpolarization-activated cyclic nucleotide-gated (HCN) channels in neuronal excitability and synaptic transmission is still unclear, it is postulated that the HCN channels may be involved in seizure activity. The aim of this study was to assess the effects of ivabradine (an HCN channel inhibitor) on the protective action of four classical antiepileptic drugs (carbamazepine, phenobarbital, phenytoin and valproate) against maximal electroshock-induced seizures in mice. Tonic seizures (maximal electroconvulsions) were evoked in adult male albino Swiss mice by an electric current (sine-wave, 25 mA, 0.2 s stimulus duration) delivered via auricular electrodes. Acute adverse-effect profiles of the combinations of ivabradine with classical antiepileptic drugs were measured in mice along with total brain antiepileptic drug concentrations. Results indicate that ivabradine (10 mg/kg, i.p.) significantly enhanced the anticonvulsant activity of valproate and considerably reduced that of phenytoin in the mouse maximal electroshock-induced seizure model. Ivabradine (10 mg/kg) had no impact on the anticonvulsant potency of carbamazepine and phenobarbital in the maximal electroshock-induced seizure test in mice. Ivabradine (10 mg/kg) significantly diminished total brain concentration of phenytoin and had no effect on total brain valproate concentration in mice. In conclusion, the enhanced anticonvulsant action of valproate by ivabradine in the mouse maximal electroshock-induced seizure model was pharmacodynamic in nature. A special attention is required when combining ivabradine with phenytoin due to a pharmacokinetic interaction and reduction of the anticonvulsant action of phenytoin in mice. The combinations of ivabradine with carbamazepine and phenobarbital were neutral from a preclinical viewpoint.

## Introduction

Ivabradine, as a hyperpolarization-activated cyclic nucleotide-gated (HCN) channel blocker inhibits directly and selectively a depolarizing mixed sodium and potassium inward current, called the “funny” current (I_f_/I_h_) in both, sino-atrial node and neurons [1–4]. Under physiological conditions, any increase or decrease in intracellular cyclic adenosine monophosphate (cAMP) level regulates the I_f_/I_h_ current, producing a shift in the voltage-dependent activation of the I_f_/I_h_ current [5]. Although the physiological role of the I_h_ current in neurons is still unclear, the I_h_ current is expected to play a pivotal role in seizure activity.

Experimental evidence indicates that ivabradine possesses the anticonvulsant properties by elevating, in a dose dependent manner, the threshold for electroconvulsions in mice and the experimentally determined TID_20_ and TID_50_ values (i.e., threshold increasing doses by 20 and 50%) for ivabradine were 8.70 and 18.29 mg/kg, respectively [6]. Since ivabradine, as an HCN channel blocker, elevates the threshold for electroconvulsions in mice, it can be expected that the drug will also be able to enhance the anticonvulsant potency of some classical antiepileptic drugs. Thus, a favorable effect observed for ivabradine in combination with classical antiepileptic drugs in experimental animals could significantly increase our knowledge about neuronal mechanisms involved in seizure initiation, propagation and amplification in the brain, especially, in these brain regions where I_h_ currents are abundantly distributed in neurons.

The aim of this study was to assess the influence of ivabradine on the protective action of four classical antiepileptic drugs (carbamazepine, phenytoin, phenobarbital and valproate) in the mouse maximal electroshock-induced tonic seizure model. Generally, in the maximal electroshock seizure test it is possible to determine whether ivabradine was able to enhance or alleviate the anticonvulsant potency of the classical antiepileptic drugs in this seizure model, which is considered to be an experimental model of tonic-clonic seizures and partial convulsions with or without secondary generalization in humans [7]. Additionally, the effects of ivabradine alone and in combination with the classical antiepileptic drugs were examined in three behavioral (chimney, passive avoidance, and grip-strength) tests to detect any possible impairment of motor coordination, disturbances in long-term memory, and changes in skeletal muscular strength in animals. To exclude any pharmacokinetic interactions for the observed effects in the mouse maximal electroshock-induced seizure model between ivabradine and the classical antiepileptic drugs, total brain antiepileptic drug concentrations were measured with fluorescence polarization immunoassay.

## Materials and Methods

### Animals

Adult male Albino Swiss mice (weighing 22–26 g), after 7 days of adaptation to laboratory conditions, were randomly assigned to experimental groups, each comprised 8 mice. All procedures involving animals and their care, described in this study, were approved by the First Local Ethics Committee for Animal Experiments at the Medical University of Lublin (License No.: 13/2015), and complied with the European Communities Council Directive of 24 November 1986 (86/609/EEC). All experiments on animals described below are in accordance with ARRIVE guidelines. Total number of animals used in this study was 320.

### Drugs

Ivabradine (Procoralan®, Les Laboratoires Servier, Neuilly-sur-Seine, France), carbamazepine (Polpharma, Starogard Gdański, Poland), phenobarbital (Polfa, Kraków, Poland), phenytoin (Polfa, Warszawa, Poland) were suspended in a 1% solution of Tween 80 (Sigma-Aldrich) in distilled water. Only valproate (sodium salt—Sigma-Aldrich) was directly dissolved in distilled water. All drugs were administered intraperitoneally (*ip*) as follows: phenytoin—120 min, ivabradine and phenobarbital—60 min, carbamazepine and valproate—30 min before maximal electroshock-induced seizures, behavioral (passive-avoidance, grip-strength and chimney) tests and before collection of brains for the measurement of antiepileptic drug concentrations, as documented earlier [6, 8–10].

### Maximal Electroshock Seizure Test

In the maximal electroshock seizure test, mice were challenged with a current (sine-wave, 25 mA, 50 Hz, 500 V, stimulus duration 0.2 s) delivered via ear-clip electrodes from a rodent shocker generator (Hugo Sachs Elektronik, Freiburg, Germany). The tonic hind limb extension in mice was the endpoint. The classical antiepileptic drugs administered singly and their combination with ivabradine were tested for their ability to increase the number of mice protected from maximal electroconvulsions. Log-probit method [11] was used to determine median effective dose (ED_50_) values for the antiepileptic drugs tested. The mice were injected with ivabradine in doses of 5 and 10 mg/kg that by themselves did not significantly affect the threshold for electroconvulsions [6]. Total number of animals used in this procedure was 240.

### Measurement of Total Brain Antiepileptic Drug Concentrations

The measurement of total brain concentrations of phenytoin and valproate (at doses that corresponded to their ED_50_ values from the maximal electroshock seizure test) was performed by a fluorescence polarization immunoassay. The mice received a given antiepileptic drug alone and in combination with ivabradine (10 mg/kg), and subsequently, the mice were decapitated. The whole brains of mice were removed from the skulls, weighed, harvested and homogenized using Abbott buffer (1:2, w/v; Abbott Laboratories, North Chicago, IL, USA). The homogenates were centrifuged at 10,000×*g* for 10 min and the supernatant samples of 100 μl were collected and then analyzed for phenytoin or valproate content by Abbott TDx analyzer, as described earlier [12]. Total brain phenytoin and valproate concentrations are expressed in μg/g of wet brain tissue as the means ± S.E.M. (n = 8 mice per group). Total number of animals used in this procedure was 32.

### Step-Through Passive Avoidance Task

The assessment of any acute adverse effect potential of ivabradine (10 mg/kg) alone and in combination with classical antiepileptic drugs (at doses that corresponded to their ED_50_ values from the maximal electroshock seizure test) with respect to deficits in long-term memory in mice was quantified by the step-through passive avoidance task, as described earlier [8, 9, 13]. Long-term memory in mice is expressed as the median latencies (retention times) with 25th and 75th percentiles (n = 8 mice per group). Total number of animals used in this procedure was 48.

### Grip-Strength Test

The assessment of any acute adverse effect potential of ivabradine (10 mg/kg) alone and in combination with classical antiepileptic drugs (at doses that corresponded to their ED_50_ values from the maximal electroshock seizure test) with respect to changes in skeletal muscular strength in mice was quantified by the grip-strength test, as published elsewhere [8, 9, 14]. The skeletal muscular strength in mice is expressed in newtons (N) as the means ± S.E.M. (n = 8 mice per group). Total number of animals used in this procedure was 48.

### Chimney Test

The assessment of any acute adverse effect potential of ivabradine (10 mg/kg) alone and in combination with classical antiepileptic drugs (at doses that corresponded to their ED_50_ values from the maximal electroshock seizure test) with respect to impairment of motor coordination in mice was performed by the use of the chimney test, as described earlier [8, 9, 15]. Results from the chimney test are expressed as percentage (%) of animals showing motor coordination impairment (n = 8 mice per group). Total number of animals used in this procedure was 48.

### Statistics

Statistical analysis of data from the maximal electroshock seizure test was performed either with the log-probit method [11] for single comparisons between two ED_50_ values, or with one-way ANOVA, followed by the *post-hoc* Tukey–Kramer test for multiple comparisons among three ED_50_ values, as described earlier [16]. The unpaired Student’s *t* test was used to statistically compare total brain antiepileptic drug concentrations. The Fisher’s exact probability test was used to analyze qualitative variables from the chimney test. The Kruskal–Wallis nonparametric ANOVA analyzed data from the passive avoidance task, whereas the results from the grip-strength test were statistically verified with one-way ANOVA. Statistical significance was established at P < 0.05.

## Results

### Influence of Ivabradine on the Protective Activity of Carbamazepine, Phenobarbital, Phenytoin and Valproate in the Mouse Maximal Electroshock Seizure Model

**Fig. 1:**
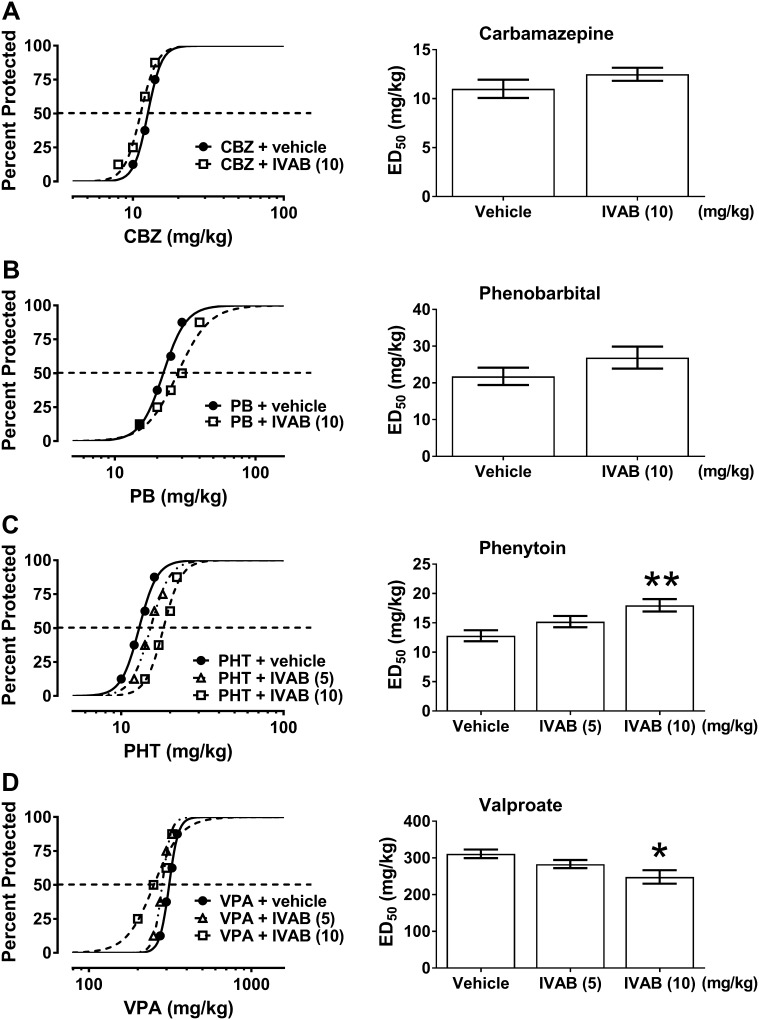
**a**–**d** Effect of ivabradine (IVAB) on the protective activity of carbamazepine (CBZ), phenobarbital (PB), phenytoin (PHT), and valproate (VPA) against maximal electroshock-induced seizures in mice. *Left panel* Dose–response function for the anticonvulsant (protective) activity of classical antiepileptic drugs [CBZ (**a**), PB (**b**), PHT (**c**) and VPA (**d**)] alone and in combination with ivabradine (IVAB) in the mouse maximal electroshock-induced seizure model. Each data point represents percent of mice protected (n = 8 mice/data point) from maximal electroshock-induced seizures at a given dose (in mg/kg). Sigmoidal curves are the result of a least squares fit of dose–response function for each antiepileptic drug. Points of intersections with the dashed line at 50% correspond to approximate ED_50_ values of antiepileptic drugs. *Right panel* Columns represent median effective doses (ED_50_ in mg/kg ± S.E.M.) of antiepileptic drugs that protect 50% of animals tested against maximal electroshock-induced seizures in mice. The log-probit method was used for calculating the ED_50_ values and statistical analysis of data was performed either with log-probit method for single comparison (**a, b**) or with one-way ANOVA followed by the post-hoc Tukey–Kramer test for multiple comparisons (**c, d**). *P < 0.05 and **P < 0.01 versus control (antiepileptic drug + vehicle-treated) animals

Ivabradine (10 mg/kg, i.p.) significantly reduced the anticonvulsant potency of phenytoin by increasing its ED_50_ value from 12.80 ± 0.94 to 17.99 ± 1.07 mg/kg (P < 0.01) (F(2;61) = 5.993; P = 0.004; Fig. 1c). On the contrary, ivabradine (10 mg/kg, i.p.) considerably potentiated the anticonvulsant action of valproate by decreasing its ED_50_ value from 311.2 ± 11.7 to 248.2 ± 18.3 mg/kg (P < 0.05) (F(2;61) = 3.456; P = 0.038; Fig. 1d). Ivabradine (5 mg/kg) had no significant impact on the anticonvulsant action of phenytoin and valproate in the mouse maximal electroshock seizure model (Fig. 1c, d). Similarly, ivabradine (10 mg/kg, ip) did not significantly alter the anticonvulsant action of carbamazepine or phenobarbital in the maximal electroshock seizure test in mice (Fig. 1a, b).

### Influence of Ivabradine on Total Brain Phenytoin and Valproate Concentrations

As measured by fluorescent polarization immunoassay method, total brain concentrations of phenytoin (18.0 mg/kg) administered separately were 2.12 ± 0.24 µg/g of wet brain tissue, whereas those of phenytoin (18.0 mg/kg) in combination with ivabradine (10 mg/kg) were significantly reduced, amounting to 1.29 ± 0.27 µg/g of wet brain tissue (P < 0.05). In contrast, ivabradine (10 mg/kg) had no significant impact on total brain concentration of valproate (248.2 mg/kg). In this case, the total brain concentrations of valproate (248.2 mg/kg) administered alone were 99.6 ± 12.2 µg/g of wet brain tissue and did not differ significantly from those for the combination of valproate (248.2 mg/kg) with ivabradine (10 mg/kg), which were 105.3 ± 11.5 µg/g of wet brain tissue.

### Effects of Ivabradine Alone and in Combination with Various Antiepileptic Drugs on Motor Performance, Long-Term Memory, and Skeletal Muscular Strength of Animals

Ivabradine (10 mg/kg, i.p.) administered alone and in combination with the studied four classical antiepileptic drugs did not disturb long-term memory, alter skeletal muscular strength or impair motor performance in mice, as determined in the passive avoidance task, grip-strength test, and chimney test, respectively (Table 1).

**Table 1:** Effects of ivabradine and its combination with four classical antiepileptic drugs on long-term memory in the passive avoidance task, motor performance in the chimney test and muscular strength in the grip-strength test in mice

Treatment (mg/kg)	Retention time (s)	Muscular strength (N)	Motor coordination impairment (%)
Vehicle	180 (180; 180)	1.087 ± 0.067	0
Ivabradine (10) + Vehicle	180 (180; 180)	1.052 ± 0.071	12.5
Carbamazepine (12.5) + Ivabradine (10)	180 (180; 180)	1.049 ± 0.065	0
Phenobarbital (26.9) + Ivabradine (10)	180 (180; 180)	1.048 ± 0.062	0
Phenytoin (18.0) + Ivabradine (10)	180 (180; 180)	1.062 ± 0.072	0
Valproate (248.2) + Ivabradine (10)	177.5 (155.5; 180)	1.063 ± 0.069	12.5

Results are presented as: median retention times [in seconds (s); with 25th and 75th percentiles in parentheses] from the passive avoidance task; mean muscular strengths [in newtons (N) ± S.E.M.] from the grip-strength test; and percentage (%) of animals with impairment of motor coordination from the chimney test. Each experimental group consisted of 8 mice

## Discussion

Results reported in this study indicate that ivabradine, as the inhibitor of HCN channels, enhanced the anticonvulsant potency of valproate against maximal electroshock-induced seizures in mice. Pharmacokinetic study revealed that the enhancement of the anticonvulsant potency of valproate (by 20%) was associated with a non-significant (6%) increase in total brain concentration of valproate in mice. Thus, it was confirmed that the observed potentiation of the anticonvulsant potency of valproate in this seizure model by ivabradine was pharmacodynamic in nature. To explain the observed potentiation of the antiseizure effects of valproate by ivabradine, one should consider molecular mechanisms of action of both drugs. Ivabradine is a drug that selectively and directly blocks the I_f_/I_h_ channels in neurons [4, 17]. Valproate blocks low-threshold T-type calcium channels, enhances GABA-ergic neurotransmission in the brain and increases the potassium-induced release of GABA in neurons. Additionally, valproate increases synthesis of GABA by activating glutamic acid decarboxylase—a GABA synthesizing enzyme. Valproate activates potassium conductance and elevates GABA levels in specific brain regions by inhibiting GABA-transaminase—an enzyme catalyzing GABA degradation (for review see: [18, 19]). Although it is difficult to comprehend the exact nature of the synergistic effect from their respective mechanisms of action, this study clearly indicates that ivabradine synergistically cooperates with valproate in terms of suppression of maximal electroshock-induced seizures.

In contrast, ivabradine did not significantly affect the anticonvulsant action of carbamazepine and phenobarbital in the mouse maximal electroshock seizure model, therefore, total brain concentrations of these antiepileptic drugs were not estimated in the presented study. On the other hand, pharmacokinetic experiments using fluorescence polarization immunoassay revealed that ivabradine considerably reduced (by 39%) total brain concentrations of phenytoin in mice, confirming that a pharmacokinetic interaction between drugs was entirely responsible for the observed reduction (by 41%) of the ED_50_ value of phenytoin in the maximal electroshock-induced seizure test in mice when combined with ivabradine. The explanation of this phenomenon may, at least in part, depend on the reduction of the heart rate in the mice exposed to the combination of phenytoin and ivabradine. Since phenytoin belongs to the subclass IB of antiarrhythmic drugs [20], and ivabradine is a specific heart rate-lowering compound [17, 21], it is possible that the combination of these two drugs may significantly reduce the heart rate in laboratory mice. Thus, it can be observed a decrease in some pharmacokinetic parameters characterizing distribution of phenytoin in mice, including, its penetration through the blood–brain barrier and dispersion of phenytoin in the brain tissue. Although this suggestion is highly speculative, it can easily explain the observed pharmacokinetic reduction in total brain concentrations of phenytoin after co-administration of ivabradine.

Previously, it was documented that phenytoin (applied in a dose of 150 mg twice daily) pharmacokinetically reduced (by approx. 70%) bioavailability of ivabradine (applied in a single dose of 10 mg) in 18 healthy volunteers [22]. Since phenytoin is an inductor of the main metabolizing enzyme of ivabradine (CYP3A4), it is highly likely that the antiepileptic drug affects pharmacokinetics of ivabradine. Of note, ivabradine is metabolized only by CYP3A4 [23], whereas phenytoin mainly by CYP2C9 and CYP2C19 [24]. Thus, the mutual induction of CYP isoenzymes should be borne in mind while explaining the observed pharmacokinetic reduction in total brain phenytoin concentration in experimental animals receiving ivabradine. On the other hand, valproate is also metabolized by CYP2C9 [24], and ivabradine as an inducer of CYP2C9 should also reduce valproate concentrations in animals. Since no significant changes in valproate concentrations were observed in the mouse brain tissue, this pharmacokinetic mechanism was rather not responsible for the observed effects. To thoroughly characterize pharmacokinetic parameters for the interaction between ivabradine and phenytoin, more advanced pharmacokinetic studies, based on simultaneous estimation of distribution, metabolism and elimination of both drugs are required.

In this study, we also found that ivabradine combined with four classical antiepileptic drugs, at doses corresponding to their ED_50_ values from the maximal electroshock seizure test, did not affect acute adverse effects produced by classical antiepileptic drugs in the chimney, step-through passive avoidance and grip-strength tests in mice. Results from these behavioral tests may indirectly suggest that the heart rate-lowering effect of the combination of phenytoin with ivabradine was not so meaningful, permitting the animals to correctly perform all the studied behavioral tests without any significant impairment in locomotor activity, learning and skeletal muscular strength in mice. The similar situation was documented in animals receiving ivabradine in combination with carbamazepine, phenobarbital and valproate.

## Conclusion

The application of ivabradine together with valproate may be clinically favorable due to the pharmacodynamic enhancement of the anticonvulsant potency of the latter drug. In contrast, a special attention is advised to patients receiving phenytoin with ivabradine because of pharmacokinetic reduction of phenytoin concentrations that may result in alleviation of protection of the antiepileptic drug from seizures. In the case of the combinations of ivabradine with carbamazepine or phenobarbital, no significant changes in the anticonvulsant properties of the studied antiepileptic drugs are expected. If the results from this preclinical study could be translated to clinical settings, ivabradine would be combined with classical antiepileptic drugs, except for phenytoin.
